# Dietary salt exacerbates intestinal fibrosis in chronic TNBS colitis via fibroblasts activation

**DOI:** 10.1038/s41598-021-94280-8

**Published:** 2021-07-23

**Authors:** Asma Amamou, Matthieu Rouland, Linda Yaker, Alexis Goichon, Charlène Guérin, Moutaz Aziz, Guillaume Savoye, Rachel Marion-Letellier

**Affiliations:** 1 Normandie Univ, INSERM Unit 1073, University of Rouen Normandy, Rouen, France; 2 Institute for Research and Innovation in Biomedicine (IRIB), University of Rouen Normandy, Rouen, France; 3 Anatomopathology Department, Rouen University Hospital, Rouen, France; 4 Gastroenterology Department, Rouen University Hospital, Rouen, France

**Keywords:** Gastrointestinal diseases, Preclinical research

## Abstract

Intestinal fibrosis is a frequent complication in inflammatory bowel diseases (IBD). It is a challenge to identify environmental factors such as diet that may be driving this risk. Intestinal fibrosis result from accumulation of extracellular matrix (ECM) proteins secreted by myofibroblasts. Factors promoting intestinal fibrosis are unknown, but diet appears to be a critical component in its development. Consumption of salt above nutritional recommendations can exacerbate chronic inflammation. So far, high salt diet (HSD) have not been thoroughly investigated in the context of intestinal fibrosis associated to IBD. In the present study, we analyze the role of dietary salt in TNBS chronic colitis induced in rat, an intestinal fibrosis model, or in human colon fibroblast cells. Here, we have shown that high-salt diet exacerbates undernutrition and promoted ECM-associated proteins in fibroblasts. Taken together, our results suggested that dietary salt can activate intestinal fibroblasts, thereby contributing to exacerbation of intestinal fibrosis. Dietary salt may be considered as a putative environmental factor that drives intestinal fibrosis risk.

## Introduction

The natural history of Inflammatory bowel diseases (IBD) is frequently complicated by intestinal fibrosis and formation of narrowing of the lumen called strictures. This is the case for more than 30% of Crohn’s disease (CD) and 5% of ulcerative colitis^1^. It has been previously shown that stricturing CD may not well respond to TNF inhibition^2,3^. Intestinal fibrosis leads to surgery in CD and strictures frequently recur leading to repeated surgeries. Chronic mucosal inflammation induces fibrosis by a cascade of events from intestinal epithelial damages to angiogenesis, immune and mesenchymal cells activation^4^. Ileal genes controlling extracellular matrix production are upregulated at diagnosis in pediatric CD and this is associated with stricturing^3^. It is thus important to identify environmental factors that may be driving this risk. There is currently no specific therapy to prevent or inhibit intestinal fibrosis and this therefore constitutes a major treatment challenge.

Factors promoting intestinal fibrosis are currently unknown but diet is a potential culprit. First, diet is the most common patient-reported triggers of disease activity^5^. Western diet pattern is associated with increased IBD incidence^5^ and incidence of IBD continues to increase in the newly industrialized countries adopting Western diet^6^. Diet plays a key role in controlling gut and immune homeostasis^7^. Even IBD patients in remission have considerably distorted and unhealthy dietary intake with an exceeded dietary intake for sodium^8^. Sodium intakes around the world are higher of physiological need (i.e. 10–20 mmol/day) and most adult populations have mean sodium intakes > 100 mmol/day^9^. In Northern American and European countries, sodium intake is mainly provided by sodium added in processed foods (approximately 75% of intake)^9^. Few recent studies have shown the potential of dietary salt to promote intestinal inflammation in colitis models^10–12^. It thus raised the potential role of dietary salt to induce a more vulnerable environment to inflammatory insults.

As the impact of dietary salt on intestinal fibrosis has not been studied, we aimed to investigate its effects on chronic colitis and fibrosis.

## Results

### Dietary salt promotes undernutrition in chronic colitis

**Figure 1:**
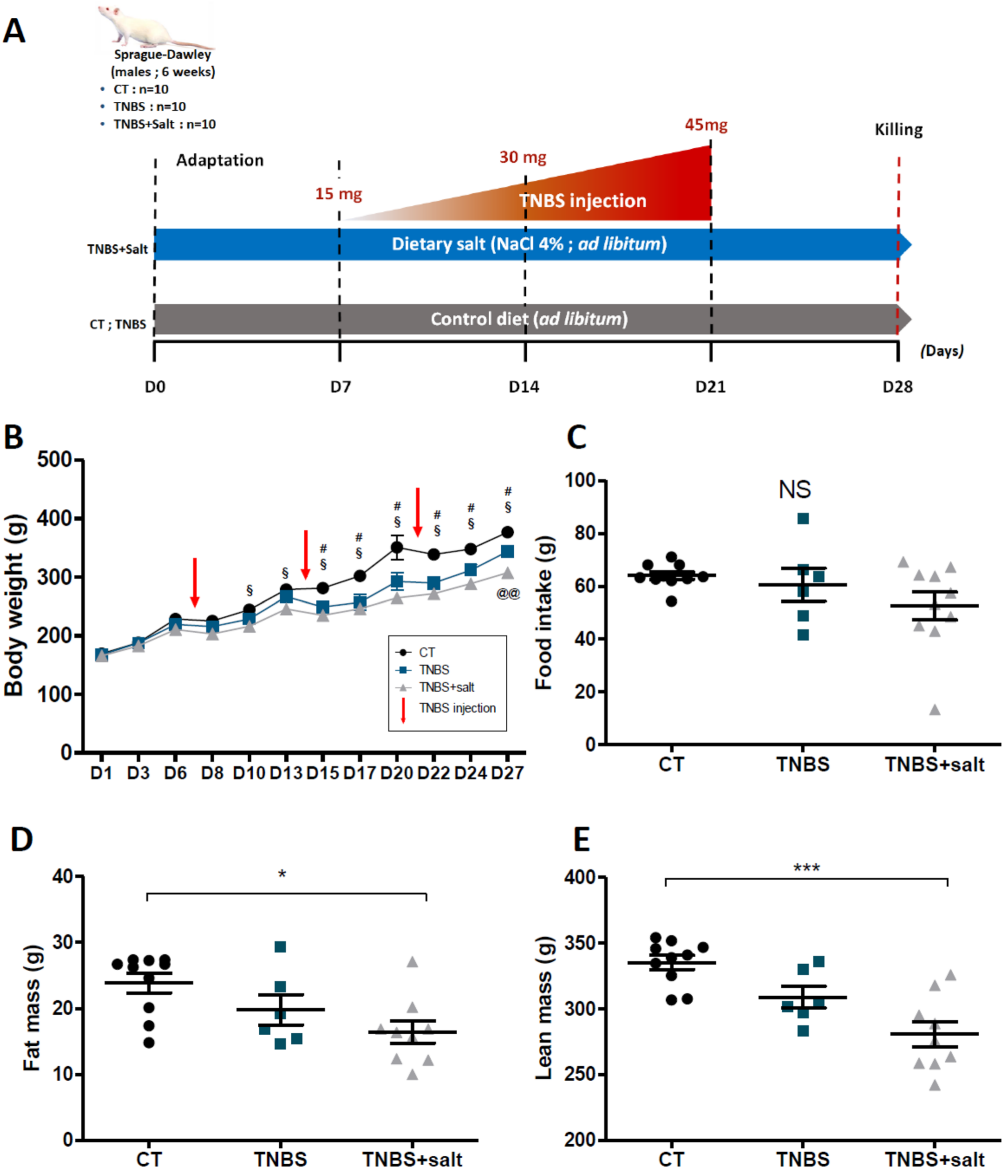
Dietary salt exacerbates undernutrition in rats with chronic colitis. Male Sprague–Dawley rats underwent weekly trinitrobenzene sulfonic acid (TNBS; n = 10) enemas for 3 weeks to induce chronic colitis-induced intestinal fibrosis, whereas control rats received a saline solution (CT; n = 10). Rats were subjected to either a standard diet or high-salt diet (4%, w/w) for 4 weeks (TNBS + salt, n = 10). (**A**) Experimental design. (**B**) Body weight. (**C**) Food intake. Body composition by echoMRI measurement: (**D**) fat mass and (**E**) lean mass. Control (CT, n = 10); TNBS (n = 6); TNBS + salt (n = 9). (**B**) Two-way ANOVA followed Bonferroni post-test: §means p < 0.05: CT vs TNBS + salt, #p < 0.05: CT vs TNBS, @@p < 0.01: TNBS vs TNBS + salt. (**C**,**D**,**E**) Kruskal–Wallis test followed post-test de Dunn: *p < 0.05, ***p < 0.001 respectively.

We thought whether high-salt diet promotes intestinal fibrosis. To test this, TNBS-chemically induced chronic colitis was carried out in rats and we examined the effects of high-salt diet (NaCl 4%) (TNBS + Salt) on fibrosis development (Fig. 1A). Colitis induction significantly decreased body weight in TNBS and TNBS + Salt groups compared to CT group from the first TNBS instillation until the end of the experiment. At the end of the protocol, TNBS + Salt rats exhibited a lower body weight compared to TNBS rats fed with a standard diet (Fig. 1B). To measure food intake, animals were placed in a metabolic cage for 48 h. No difference of food intake between groups was found. At 27th day, body composition was measured by EchoMRI. While CT and TNBS rats had no difference of lean and fat mass, TNBS + Salt exhibited a significative reduced proportion of lean and fat mass compared to the CT group (Fig. 1C,D,E).

### Dietary salt stimulates intestinal inflammation in chronic colitis

**Figure 2:**
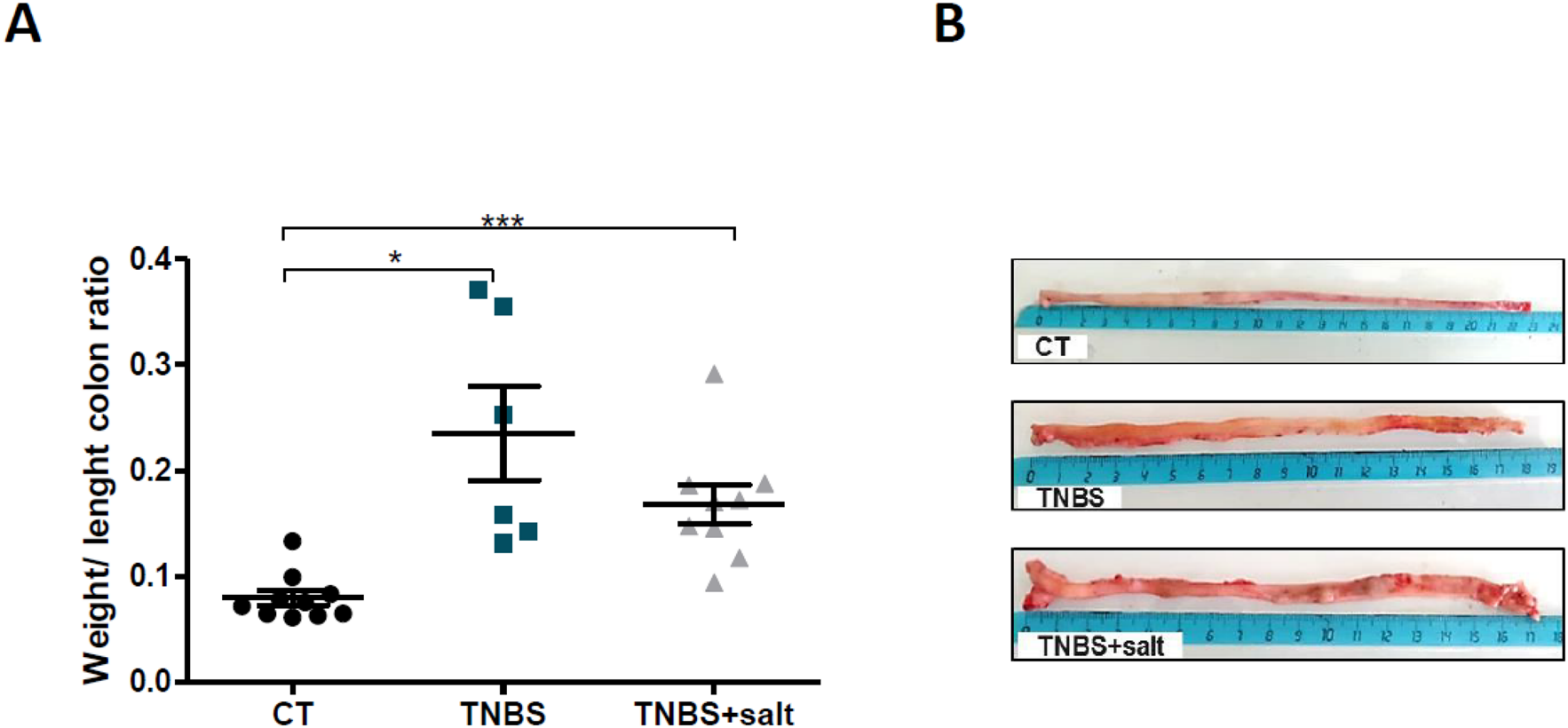
Colon weight/length ratio in chronic colitis. Male Sprague–Dawley rats underwent weekly trinitrobenzene sulfonic acid (TNBS; n = 10) enemas for 3 weeks to induce chronic colitis-induced intestinal fibrosis, whereas control rats received a saline solution (CT; n = 10). Rats were subjected to either a standard diet or high-salt diet (4%, w/w) for 4 weeks (TNBS + salt, n = 10). (**A**) Colon weight/length ratio. (**B**) Representative images of rat colon. Control (CT, n = 10); TNBS (n = 6); TNBS + salt (n = 9). One way ANOVA followed Tukey post-test: * means p < 0.05, ***p < 0.001 respectively.

Colon weight/Length ratio was commonly used as an inflammatory marker^13–15^ and this ratio was significantly higher for TNBS and TNBS + salt groups compared to CT group (Fig. 2A,B). Similarly, inflammatory score is higher in TNBS and TNBS + salt groups (1.833 ± 0.4014, 2.125 ± 0.3504 respectively) compared to CT group (0.1000 ± 0.1000; P < 0.0001 vs CT) (Supplementary Fig. 1D).

### Dietary salt promotes intestinal fibrosis in chronic colitis

**Figure 3:**
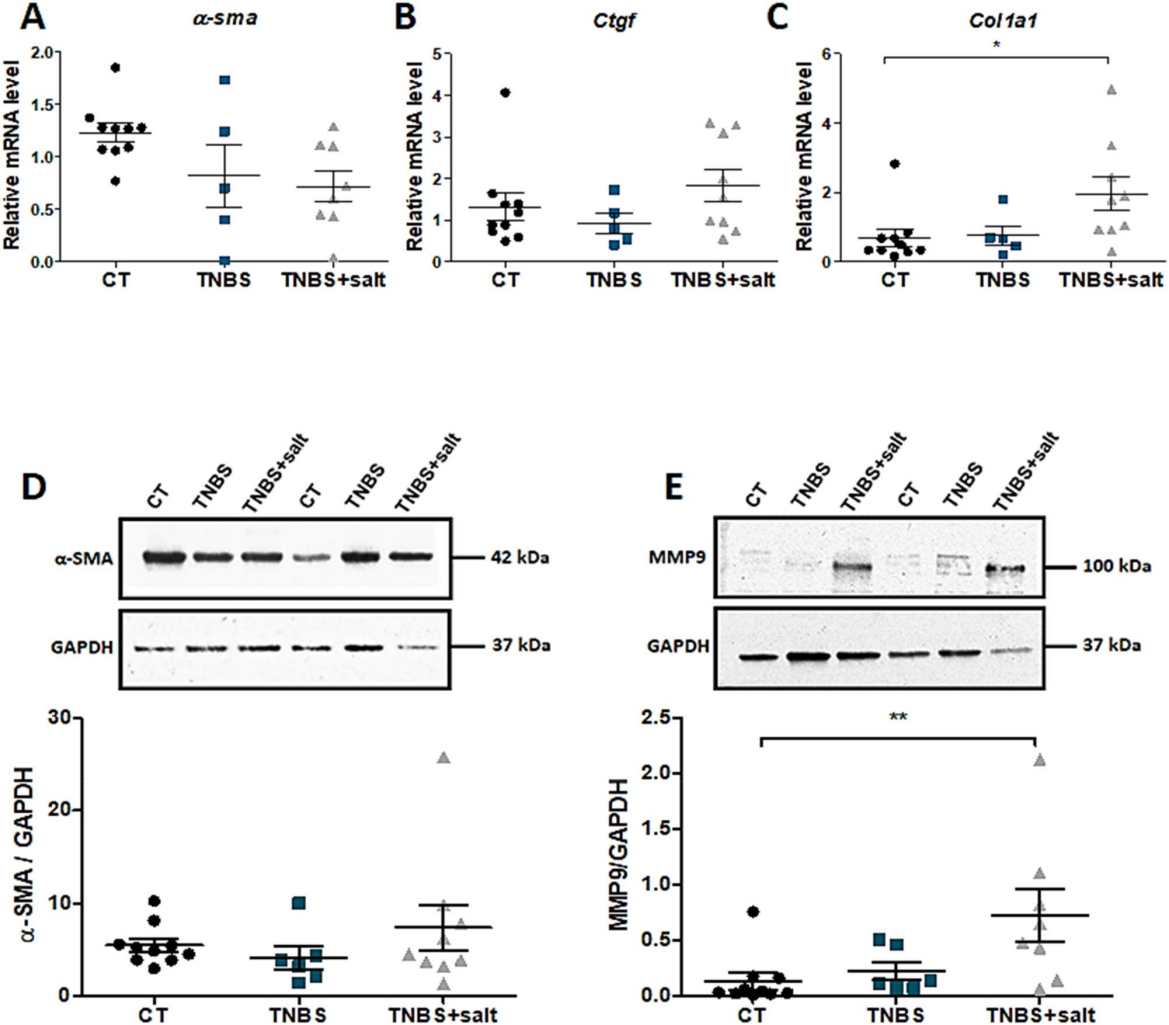
Dietary salt promotes collagen 1 and MMP-9 in chronic colitis-induced intestinal fibrosis. Male Sprague–Dawley rats underwent weekly trinitrobenzene sulfonic acid (TNBS; n = 10) enemas for 3 weeks to induce chronic colitis-induced intestinal fibrosis, whereas control rats received a saline solution (CT; n = 10). Rats were subjected to either a standard diet or high-salt diet (4%, w/w) for 4 weeks (TNBS + salt, n = 10). Relative colon mRNA levels encoding for (**A**) *α-sma* (alpha smooth muscle actin), (**B**) *Ctgf* (connective tissue growth factor) and (**C**) *Col1a1* (collagen 1). Relative protein expression of colon α-SMA (**D**) and MMP-9 (**E**). GAPDH is used as an internal control. Control (CT, n = 10); TNBS (n = 6); TNBS + salt (n = 9). One-way ANOVA followed by Tukey post-test: * means p < 0.05, **p < 0.001 respectively.

**Figure 4:**
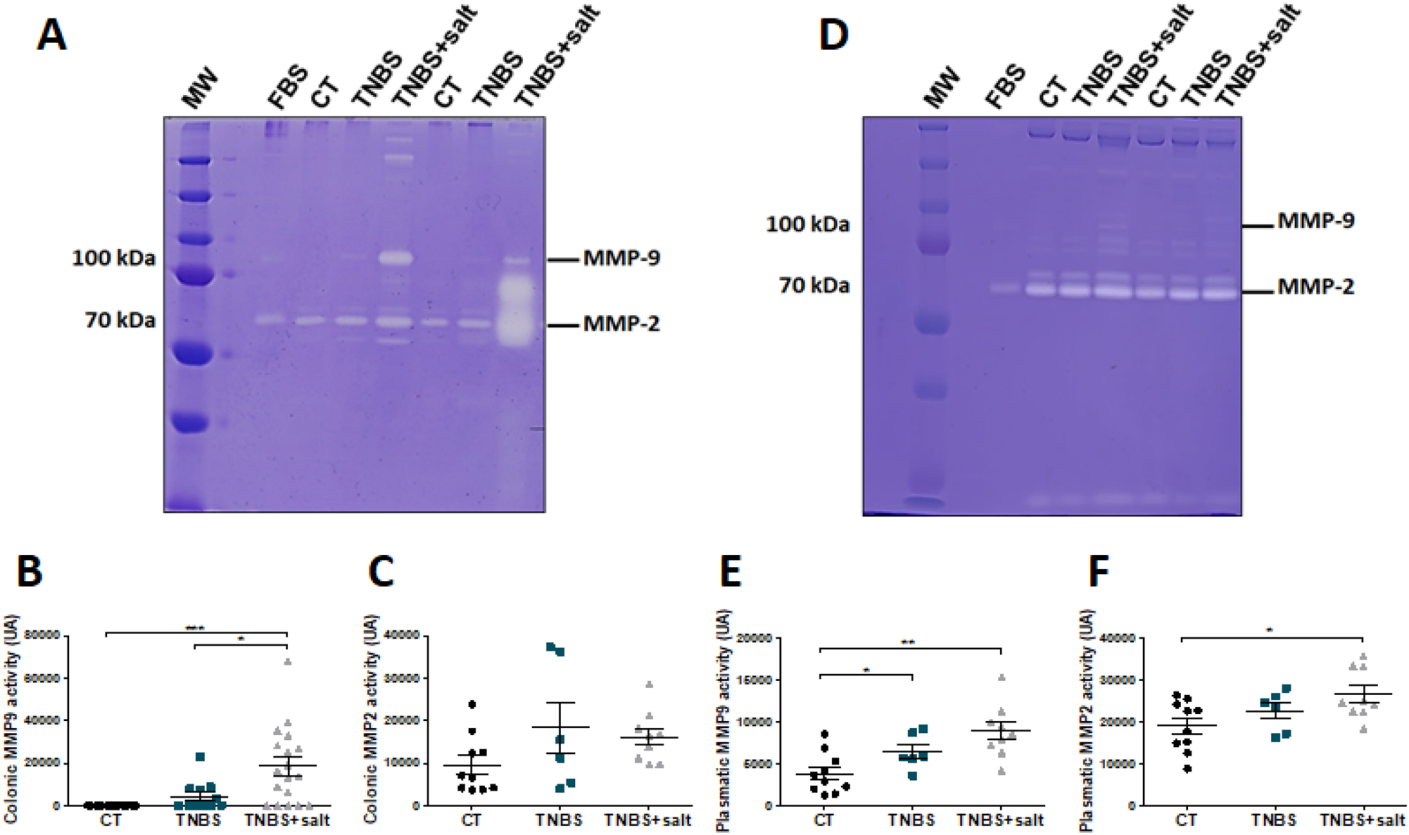
Dietary salt induces MMP-9 and MMP-2 activity in chronic colitis-induced intestinal fibrosis. Male Sprague–Dawley rats underwent weekly trinitrobenzene sulfonic acid (TNBS; n = 10) enemas for 3 weeks to induce chronic colitis-induced intestinal fibrosis, whereas control rats received a saline solution (CT; n = 10). Rats were subjected to either a standard diet or high-salt diet (4%, w/w) for 4 weeks (TNBS + salt, n = 10). (**A**) Representative gelatin zymography of colon MMP-9 and MMP-2 activity. Quantification of colon MMP-9 (**B**) and MMP-2 (**C**) activity. (**D**) Representative gelatin zymography for plasma MMP-9 and MMP-2 activity. Quantification of plasma MMP-9 (**E**) and MMP-2 (**F**) activity. Control (CT, n = 10); TNBS (n = 6); TNBS + salt (n = 9). Kruskal–Wallis test followed post-test de Dunn: *means p < 0.05, **p < 0.01, ***p < 0.001 respectively.

To assess intestinal fibrosis, fibrosis score, colon and plasmatic fibrosis markers were determined. Fibrosis score is higher in TNBS and TNBS + salt groups (1.500 ± 0.3416, 2.000 ± 0.2673 respectively) compared to CT group (0.0000 ± 0.0000; P < 0.0001 vs CT) (Supplementary Fig. 1E). Colon collagen 1 mRNA level was significantly increased in TNBS + salt group compared to CT group (p = 0.0385, Fig. 3C) while colon mRNA levels for *α-sma* and *Ctgf* did not differ among groups (Fig. 3A, B). Colon protein expression of α-SMA did not differ among groups (Fig. 3D; Supplementary Fig. 2A-B) while colon MMP-9 expression was significantly increased in TNBS + salt group compared to CT group (p = 0.0194, Fig. 3E; Supplementary Fig. 2C-D). In addition, we also determine colon and plasmatic MMP activity. We found that colon MMP-9 activity was absent in the CT group while chronic colitis induced its activity. In addition, colon MMP-9 activity was significantly up-regulated in TNBS + salt rats compared to other groups (Fig. 4A, B). No difference for colon MMP-2 activity was observed among groups (Fig. 4A, C). Plasmatic MMP-9 activity was increased in TNBS and TNBS + salt groups compared to controls (Fig. 4D, E). Plasma MMP-2 plasmatic was significantly higher in TNBS + salt group compared to CT group (Fig. 4D, F).

*NaCl treatment promotes *factors promoting IBD-induced fibrosis *in human colonic fibroblasts.*

To decipher whether NaCl may exacerbate factors promoting IBD-induced fibrosis such as myofibroblasts progenitor activation, we investigated the effects of NaCl in human colonic fibroblasts (CCD-18Co) in response to TGF-β.

**Figure 5:**
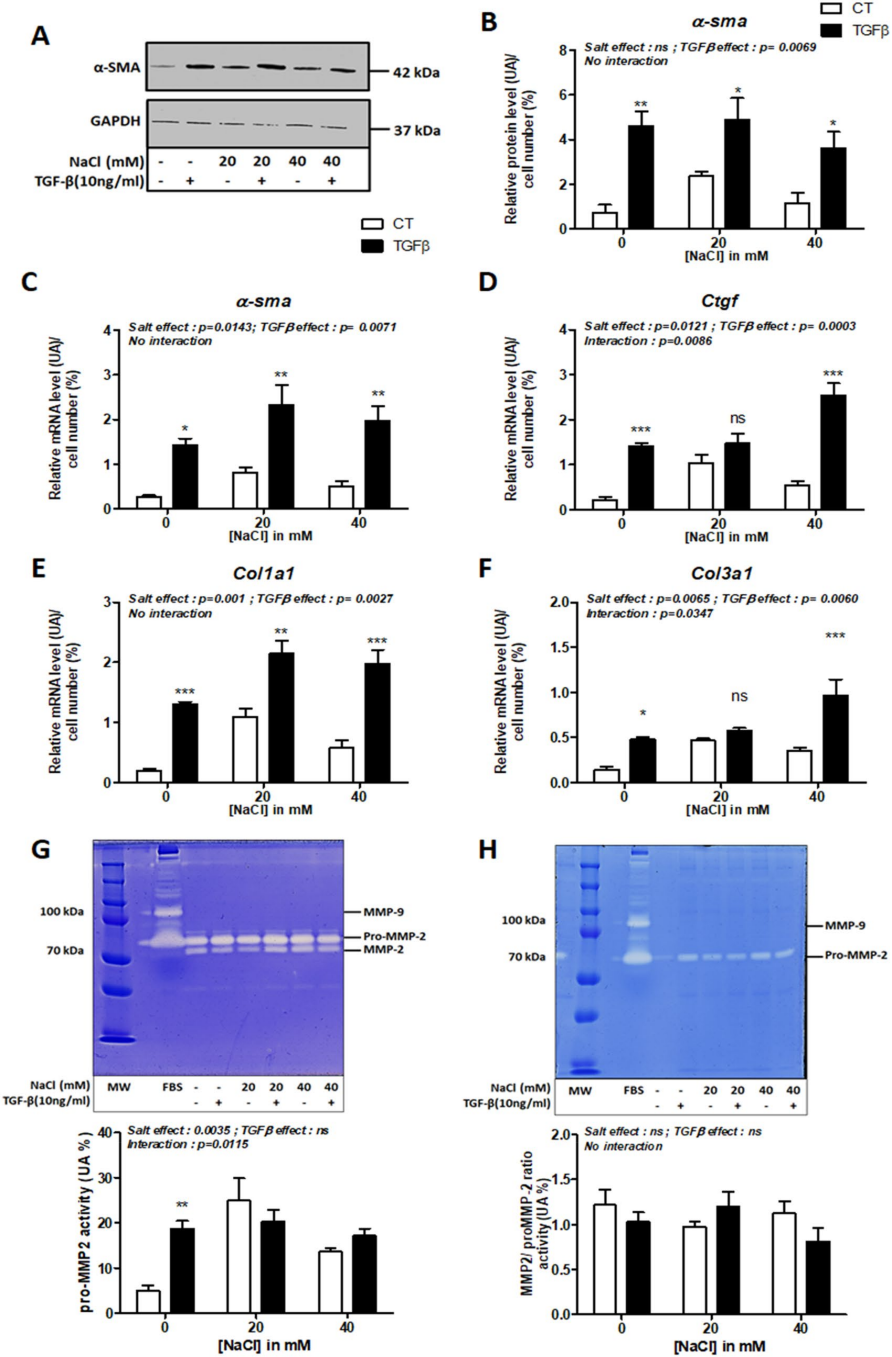
NaCl effect on CCD-18Co fibroblast cells in response to TGF-β. CCD-18Co cells were incubated with increasing concentration of dietary salt (0, 20 and 40 mM) in response to TGF-β (10 ng/mL) for 24 h. Representative western blot image (**A**) and quantification of alpha smooth muscle actin (α-SMA) in cell lysates (**B**). Relative mRNA level of *α-sma* (alpha smooth muscle actin) (**C**), *Ctgf* (connective tissue growth factor) (**D**), *Col1a1* (collagen 1) (**E**) and *Col3a1* (collagen 3) (**F**) in cell lysates. Representative gelatin zymography image or quantification of MMP-2 activity from cell supernatant (**G**) or from cell lysate (**H**). Two-way ANOVA test followed Bonferroni post-test: *means p < 0.05, **p < 0.01 respectively, ***p < 0.001.(n = 4 from independent experiments).

First, TGF-β significantly increased fibrosis markers such alpha smooth muscle actin (α-SMA) at mRNA and protein levels (p = 0.0071 and p = 0.0069 respectively) (Fig. 5A,B,C; Supplementary Fig. 3A-B), and mRNA levels for Connective tissue growth factor (*Ctgf*, p = 0.0003), collagen 1 (*Col1a1*, p = 0.0027) and 3 (*Col3a1*, p = 0.0060) (Fig. 5D,E,F). NaCl treatment significantly upregulated mRNA levels for *α-sma*, *Ctgf*, *Col1a1* and *Col3a1* (Fig. 5C,D,E,F). We had also found a significate interaction between salt and TGF-β effects for *Ctgf* (p = 0.0086) and *Col3a1* (p = 0.0347) mRNA expression (Fig. 5D,F). MMPs activity was also analysed by gelatin zymography from CCD-18Co cells supernatant and lysates. In cells supernatant, TGF-β or salt treatment did not modulate pro-MMP-2 or MMP-2 activity (Fig. 5G). From cells lysates, MMP-9 activity remained undetectable (Fig. 5H). However, in absence of NaCl, TGF-β treatment increased pro-MMP2 activity (Fig. 5G). CCD-18Co activated by TGF-β and treated with salt did not significantly modulate pro-MMP-2 activities while NaCl treatment had a significative impact on pro-MMP2 activity (p = 0.0035, Fig. 5H).

## Discussion

Environmental factors such as diet may influence predisposition to develop inflammatory bowel disease (IBD)^16^ or alter its natural history by modification of the host immune response. Intestinal fibrosis is a common IBD complication and mechanisms underlying fibrogenesis in chronic colitis are largely unknown. There is thus an urgent need for identification of dietary targets to prevent or limit intestinal fibrosis. Indeed, Western diet promotes fibrosis in studies from other organs^17,18^. Western diet is characterized by an insufficient intake of healthy foodstuffs and an excessive amount of saturated fats, sugar and salt. Processed food is a high provider of dietary sodium chloride (NaCl). Among dietary factors, the specific effect of dietary salt on IBD outcomes is poorly documented compared to the effects of sugar or other western diet components^19^. Few recent studies have evaluated the potential impact of dietary salt in colitis models^20–22^ but its effect on intestinal fibrosis has never been specifically studied.

We used a chemically induced-chronic colitis to induce intestinal fibrosis^13^. Previously, this chronic TNBS model with relapse and remission was developed in order to reproduce the disease course of human IBD^23^. Rats received 4% NaCl, a concentration similar to that present in western diet^12,24^.

In human studies, undernutrition is defined by BMI, weight loss and plasma albumin. The most important clinical indicator is the weight, in particular the speed of weight loss. By contrast, plasma albumin is not a good indicator in inflammatory states. Indeed, it has been demonstrated in IBD patients that a low serum albumin is likely a marker of systemic inflammation^25^. As serum albumin is a negative acute phase reactant, its plasmatic level decreases in proinflammatory states. More recently, low fat free mass index has been validated as a diagnostic criterion for undernutrition in human studies in European guidelines^26^. The determination of body mass composition by EchoMRI is thus a technique of choice for nutritional assessment in colitis models. Chronic TNBS induced body weight loss as previously^14^ and undernutrition is often observed in active IBD patients^27^. A high-salt diet consumption exacerbates body weight loss and higher body weight loss has been observed in acute colitis mice fed with a high-salt diet^10,28^. A high-salt diet in rats also leads to a body composition alteration with a decreased fat and lean mass in colitic rats without any modification in food intake. These data suggest that high-salt diet promotes undernutrition and IBD patients are already at increased risk of malnutrition^27^. Of note, undernutrition is associated with higher complications in IBD patients such as hospitalization, longer admission or increased mortality^27^.

As previously^13–15^, TNBS colitic rats had a higher colon weight/length ratio, a surrogate marker for intestinal inflammation. When compared to colitic rats, we had a similar colon weight/length ratio in colitis rats fed with a high-salt diet. By contrast, Wei et al*.* observed a higher colon weight in high-salt diet-fed mice with acute TNBS colitis compared to standard diet-fed mice and it may be dependent of the time between the last TNBS administration and the killing: 3 days for Wei study instead of 7 days for ours^28^.

Chronic TNBS did not upregulate mRNA levels for Extra-cellular matrix (ECM) associated proteins *Col1a1* and *α-sma*. We previously observed upregulation of α-SMA protein in chronic TNBS colitis^15^ and this discrepancy may be explained by the number of TNBS administration three instead of 6 in the former protocol^15^. Even if α-SMA is usually a gold standard for fibrosis, previous studies did not observe increased α-SMA^29^ while their animal exhibited high collagen deposition and a high colon TGF-β1 level. In addition, it may be explained because Th1 and Th17 pro-inflammatory cytokines, which are associated to chronic inflammation, are able to down-regulate colon α-SMA expression^30^ in colonic subepithelial myofibroblasts isolated from healthy individuals. A fine balance between ECM production and degradation is maintained by matrix metalloproteinases (MMPs) and this balance seems to be altered in IBD. MMP-2 and MMP-9 are upregulated during IBD. MMPs, by degrading ECM, promote cell migration and the release and/or activation of growth factors and cytokines. Several studies have highlighted the role of MMPs in digestive diseases and currently pave the way to new therapeutic perspectives. In addition, direct implication of MMPs including MMP-2 and MMP-9 in the pathophysiology of IBD was supported by many studies^31^. In the present study, chronic TNBS increased plasma and colon MMP-9 activity and these results are in accordance with Garg *et* al. The authors of this study found colon up-regulation of MMP-2 and MMP-9 expression in mice with chemically-induced colitis or *Salmonella typhimurium-*induced colitis^32^.

High-salt diet promotes fibrogenesis in rats with chronic TNBS by increasing colon MMP-9 activity, plasma MMP-2 activity and colon *Col1a1* mRNA expression. This effect was not yet documented in the gut but this finding is in accordance with a study in cardiac fibroblasts^33^. A high-salt diet consumption (NaCl 8%) up-regulated mRNA levels of ECM-associated proteins such as MMP-9 and collagen I in heart fibroblasts from hypersensitive rats^33^. Dietary salt may promote intestinal fibrosis by creating a deleterious environment leading to a gut more vulnerable to inflammatory insults. Indeed, dietary salt may have a role in shaping fibroblast function. Interestingly, Aguiar *et* al. reported that dietary salt exacerbated colitis but salt by itself can trigger gut inflammation by increasing intestinal permeability and inflammatory histological score^11^. Indeed, dietary salt by itself (4%) for 3 weeks impacts gut barrier function^11^. Histological assessment of intestinal wall showed depletion of goblet cells and a higher histological inflammatory score^11^ without inducing colitis. More recently, high-salt diet also has a deleterious impact on intestinal microbiota by decreasing *lactobacillus* levels and short chain fatty acid production^20^. Interestingly, deleterious effect of high-salt diet on colitis development was absent in germ-free mice^20^.

Fibroblasts belong to mesenchymal cell population. Upon inflammatory stimuli, activated mesenchymal cell acquired myofibroblast phenotype, whereby fibroblasts transform into myofibroblasts acquiring smooth muscle features (ie α-SMA expression) and ECM synthesis. Myofibroblasts are thus an intermediate phenotype between fibroblasts (ECM production) and smooth muscle cells (contractility)^34^. Myofibroblast activation is a common feature in intestinal fibrosis and mesenchymal cells can be activated through multiple pathways. Since the main progenitor cells of activated myofibroblasts, we have evaluated in vitro NaCl treatment in TGF-β -activated human intestinal fibroblast cell line CCD-18Co. TGF-β1 is a key cytokine in intestinal fibrosis. TGF-β1 differentiates fibroblasts to myofibroblasts via upregulation of α-SMA expression. TGF-β1 also activated CTGF to promote fibroblast proliferation and production of ECM proteins such as collagen and matrix metalloproteinases (MMP). Fibrosis process is dependent on the balance between the production and degradation of ECM proteins. ECM degradation is mediated by MMP. We found that TGF-β1 increases expression of mRNA levels of ECM-associated proteins in CCD-18co myofibroblast cells such as α-SMA, CTGF, collagen I (COL1A1), and collagen III (COL3A1). CTGF and α-SMA expression are upregulated in Crohn’s disease patient fibroblasts^35,36^. It is widely accepted that TGF-β1 plays a crucial role in tissue fibrosis by inducing ECM production in fibroblasts. Briefly, TGF-β1 directly activates Smad signalling which thus triggers pro-fibrotic gene overexpression^37^. CTGF is produced at elevated levels in the fibrotic tissues^35^ and stimulates the proliferation of fibroblasts as well as the production of EMC proteins (*i.e.* COL1A1 and COL3A1)^38^. These results suggest that CCD-18Co cells have developed an activated myofibroblastic phenotype after TGF-β stimulation as previously shown by others^39^. The increased mRNA level expression of ECM-associated proteins such as α-SMA, CTGF, COL1A1 and COL3A1 was also observed by NaCl treatment. Many studies have shown a profibrotic role of dietary salt, from extra-intestinal organs, in particular in renal and heart diseases^40,41^. To further characterize the cellular effects of salt, we also analysed the expression and activity of MMPs. In our study, both TGF-β and salt increases pro-MMP-2 activity in cell lysis. However, no significant difference was found in MMP-2 activity in cell supernatant. A drastic up-regulation of MMP-2 activity after incubation of high-salt concentrations has already been highlighted in both in vitro and in vivo studies^42^. It is also worth noting that the MMP-9 activity was not detectable in our in vitro study. MMP-9 plays a crucial role in inflammatory and fibrotic diseases. However, the absence of MMP-9 activity for benefit of MMP-2 activity has been found in vitro in different types of fibroblasts such as renal and dermal fibroblasts^43^.

A better understanding of how and which environmental factors such as diet enhance the risk of intestinal fibrosis is mandatory and our experimental data highlight the potential of dietary salt in promoting undernutrition and colon fibroblast activation (graphical abstract). Clinical trials are now required to confirm our data and obtain further evidence to provide novel dietetic recommendations to IBD patients with a scientific rationale.

In conclusion, our study shows that high-salt diet can activate intestinal fibroblasts, thereby contributing to exacerbation of intestinal fibrosis.

## Material and methods

### Cell culture and treatment

Human colon fibroblast cell line CCD-18Co was purchased from ATCC (USA) and used between passages 4 to 10. Cells were grown in Minimum Essential Medium Eagle (Eurobio, France) supplemented with 10% of fetal bovine serum (FBS, Gibco, USA), 1% of penicillin/streptomycin (Dutscher, France), 2 mM of L-glutamine (Dutscher, France) and 1% of MEM non-essential amino acid (Sigma, USA). Cells were maintained in a 5% CO_2_ air‐humidified atmosphere at 37 °C. Cells were plated at a density of 150 000 cells/ 90 mm diametric petri dish (ThermoFisher, USA). The culture medium was refreshed every second day. For cell induction, CCD-18Co cells were FBS deprived 24 h before treatment. CCD-18Co cells were incubated with 10 ng/mL of TGF-β (PeproTech, USA) with increasing concentration of NaCl (0, 20 and 40 mM) for 24 h. The dose of NaCL in cell culture has been chosen on previous works investigating the effects of NaCl in intestinal inflammation^10^. After treatment, supernatants were collected and stored at − 80 °C until analysis. After three washes with PBS, cells were lysed in presence of Cellytic™ buffer (Sigma Aldrich) for protein analysis or lysed in Trizol (Invitrogen, USA) for RNA analysis. Each experiment was carried out at least 4 times. Cell number and cell viability was assessed in response to NaCl and TGF-β treatment. As NaCl decreased cell viability in a dose dependent manner (Supplementary data), cell data were normalized by respective cell number.

### Induction of colitis

Male Sprague–Dawley rats (Janvier, Le Genest-Saint-Isle, France) weighing 150 g were used. Intestinal fibrosis was induced by chronic colitis using 2,4,6-Trinitrobenzenesulfonic acid (TNBS) as previously^23^. Males Rats Sprague–Dawley were randomly divided into 3 groups: control (n = 10), TNBS (n = 10) and TNBS + salt (n = 10). In accordance to the aim of the present study and to the principles of the 3Rs, only TNBS rats were fed with high salt diet to reduce the number of used animals. Rats were food-deprived for 24 h before induction of colitis. Rats were anesthetized with an intraperitoneal injection of ketamine at 8 mg.kg^-1^ and xylazine at 2 mg.kg^-1^ following 24 h food deprivation. Rats received weekly intrarectal injections of increasing concentration of TNBS in 50% ethanol (15, 30, and 45 mg, respectively; Sigma-Aldrich, St. Louis, MO) for 3 weeks, whereas control group received the same amount of saline solution. After instillation, rats were maintained in a head-down position for a few minutes to prevent leakage of the intracolonic instillation.

### Animal diets

Rats were subjected to either standard diet (Control and TNBS rats) or a high salt diet of 4% NaCl (w/w). Control rats received standard diet while TNBS + salt rats received high salt diet (4% NaCl, SAFE, France). The dose of 4% NaCL has been chosen on previous studies investigating the effect of high salt diet in animal models^11,12,20^. Tubbs et al. calculated the NaCl content of a series of available foods in grocery and fast food restaurants using the SELF Nutrition database and the authors reported that these foods contained approximately 4% w/w NaCl^12^. Body weight and food intake were measured 3 times a week. Food intake, urinary secretion and fluid intake measurement per rat was performed for 48 h, during the experiment, using metabolic cages. Body composition was assessed by Echo-MRI-100 (Houston, USA) before rats killing.

### Killing and sampling

Rats were killed by an overdose of anesthesia the 28th day of protocol and were respectively control (n = 10, TNBS (n = 6) and TNBS + salt (n = 9). Blood was then collected, centrifuged (4 °C, 3000 rpm, 15 min) and stored at −80 °C. Colon was excised, measured weighed and divided into 1 cm pieces to be immediately nitrogen frozen and stored at -80 °C until analysis. Colon weight/length ratio or colon length measurement is a classical method to assess intestinal inflammation in colitis models^11,20,44^. We have previously demonstrated that colon weight/length is correlated to magnetic resonance colonography criteria for inflammation in rats with TNBS-induced colitis^44^.

### RT-qPCR analysis of gene expression

One-centimeter of colon tissue was homogenized in 2 mL-tubes containing 1 ml of Trizol (Invitrogen) and 200 µl of chloroform. After centrifugation at 12,000 g for 15 min at 4 °C the solution was separated into 3 distinct phases. The aqueous phase recovered was then placed with 250 μl of isopropanol. After centrifugation of the samples at 12,000 g for 10 min at 4 °C, the pellet was washed with 75% ethanol. Finally, the mRNA pellets were taken up in sterile RNAse and DNAse free water and frozen at -80° C until analysis.

First, 1 μg total RNA into cDNA by using 200 units of SuperScript™ II Reverse Transcriptase (ThermoFischer) was used for reverse transcription. SYBR™ Green technology on BioRad CFX96 real time PCR system (BioRad Laboratories, Marnes la Coquette, France) was used to perform qPCR in duplicate for each colon sample. qPCR was performed by SYBR™ for α-smooth muscle actin (*α-sma*), connective tissue growth factor (*Ctgf*), collagen 1 (*Col1a1*) and collagen 3 (*Col3a1*). *Gapdh* was used as the endogenous reference gene (ThermoFisher).

### Colon protein expression by western blot

Colon samples were homogenized in a lysis buffer containing Hepes 20 mM, KCl 300 mM, MgCl_2_ 3 mM, DTT 1 mM, EDTA 0.2 mM with 0.25% NP40, 1% of phosphatase inhibitor and 0,5% of protease inhibitor. The homogenates were centrifugated at 12,000 g for 15 min at 4 °C and the supernatant was collected. Protein concentration was determined with the Bradford method. 25 µg of protein were separated on 4–20% gradient polyacrylamide gel (Bio-Rad) by SDS-PAGE system and then transferred to a nitrocellulose membrane. For immunoblotting, membranes were blocked for 1 h at room temperature with 5% of bovine serum albumin (Eurobio, France) in Tris-buffered saline (10 mM Tris, pH = 8; 150 mM NaCl) and 0.05% Tween 20 (TBST). Membranes were then incubated overnight at 4 °C with primary antibodies: α-SMA (A5228, 1/5000, Sigma), MMP-9 (AB19016, 1/1000, Merck, Germany), GAPDH (SAB2500541, 1/5000, Sigma). After 3 TBST washes of 5 min each, membranes were incubated in appropriate secondary antibodies (1/5000, Dako, Denmark) 1 h at room temperature. Immunocomplexes were revealed by chemiluminescence detection system (GE Healthcare). Proteins bands were scanned (ImageScanner III; GE Healthcare) and analysed.

### Matrix metalloprotease (MMP) activity by gelatin zymography

MMP-2 and -9 activity was assessed by gelatin zymography. 10 µg of protein sample was diluted in a loading buffer containing: 1 M tris (pH 6.8), 2% Sodium Dodecyl Sulfate (SDS; Sigma-Aldrich), 20% glycerol (Sigma-Aldrich) and 0.02% bromophenol blue (Biorad). Samples were separated by electrophoresis on 8% (w/w) acrylamide gels containing 1% of gelatin (Sigma). After electrophoresis, gels were rinsed three times for 10 min using washing buffer (2.5% Triton X-100) at room temperature. Then, gels were incubated overnight at 37 °C with buffer solution (50 mM Tris–HCl at pH = 7.5, 5 mM CaCl2, and 0.1% Triton X-100). Gel coloration was performed with a solution containing 0.1% Blue Coomassie, 40% methanol and 10% acetic acid for 120 min. Fetal bovine serum containing MMP-9 and MMP-2 activity was used as a positive control (Gibco, USA). MMP-2 and MMP-9 activity appeared as negative staining and were quantified using ImageJ 1.51 software (NIH, USA).

### Histology

Colon samples were fixed in 4% formaldehyde and embedded in paraffin wax blocks. Sections of 4 mm were cut with a microtome and stained with hematoxylin–eosin-safran (HES) to analyzed collagen content. Epithelial necrosis, inflammatory cell infiltration and thickness of the mucosa were assessed using semi-quantitative scores that ranged from 0 to 3 for each variable (0, no inflammation; 1, very low level of inflammation; 2, moderate level of leukocyte infiltration; 3, high levels of leukocyte infiltration and vascular density, ulcerations). Fibrosis score was determined by a score ranging from 0 (no fibrosis) to 3 (severe fibrosis) depending on the density and extent of trichrome-positive connective tissue staining and disruption of tissue architecture compared with the control group. Samples were blinded and analysed with photonic VisionTek Live Digital Microscope (Sakura, The Netherland).

### Statistical analysis

Data were expressed as mean ± standard error mean and were compared using Graphpad Prism version 5.0 (Graphpad Software, La Jolla, United States). Inter-individual comparisons between two groups were performed with parametric Student *t*-test or non-parametric Mann–Whitney test, and one- or two-way ANOVA followed by Tukey or Bonferroni post-tests for more than two groups. A p value < 0.05 was considered significant.

### Ethical considerations

Animal care and experimentation complied with both French and European community regulations (Official Journal of the European Community L 358, 18/12/1986). Animal care was in accordance with institution guidelines and all experimental protocols were approved by CENOMEXA N°54. R. Marion-Letellier is authorized by the French Government to use this rat model (authorization no. N/03–11-12/26/11–15). The study was carried out in compliance with the ARRIVE guidelines.

## Supplementary Information

Supplementary Information.
