# Supplementary material for: Dietary salt exacerbates intestinal fibrosis in chronic TNBS colitis via fibroblasts activation
Source: Sci Rep. 2021 Jul 23;11:15055. doi: 10.1038/s41598-021-94280-8 (PMC8302708; doi:10.1038/s41598-021-94280-8)
Supplement: Supplementary file 1 — Supplementary Information. [file 41598_2021_94280_MOESM1_ESM.docx]

**SUPPLEMENTARY MATERIAL**

*Histology*

Colon samples were fixed in 4% formaldehyde and embedded in paraffin wax blocks. Sections of 4 mm were cut with a microtome and stained with hematoxylin-eosin-safran (HES) to analyzed collagen content. Epithelial necrosis, inflammatory cell infiltration and thickness of the mucosa were assessed using semi-quantitative scores that ranged from 0 to 3 for each variable (0, no inflammation; 1, very low level of inflammation; 2, moderate level of leukocyte infiltration; 3, high levels of leukocyte infiltration and vascular density, ulcerations). Fibrosis score was determined by a score ranging from 0 (no fibrosis) to 3 (severe fibrosis) depending on the density and extent of trichrome-positive connective tissue staining and disruption of tissue architecture compared with the control group. Samples were blinded and analysed with photonic VisionTek Live Digital Microscope (Sakura, The Netherland).


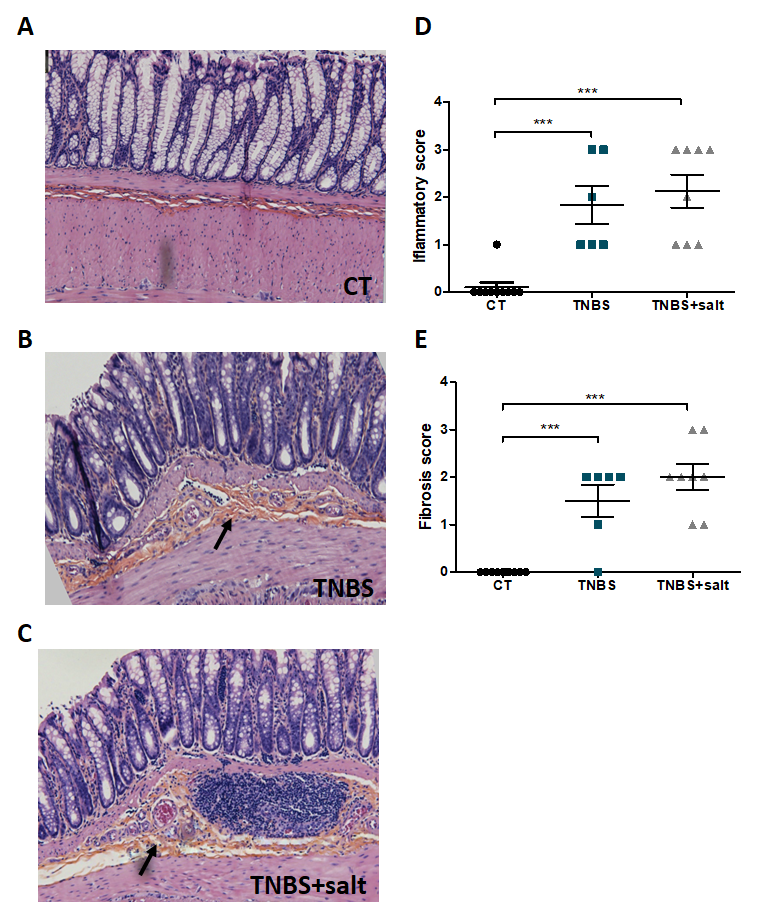


**Supplementary figure 1. Histologic inflammatory and fibrosis score from colon of chronic TNBS-induced colitis rats.**

Male Sprague-Dawley rats underwent weekly trinitrobenzene sulfonic acid (TNBS; n = 10) enemas for 3 weeks to induce chronic colitis-induced intestinal fibrosis, whereas control rats received a saline solution (CT; n=10). Rats were subjected to either a standard diet or high-salt diet (4%, w/w) for 4 weeks (TNBS+salt, n=10).

Hematoxylin-eosin-safran stained tissues in CT (**A**), TNBS(**B**) and TNBS+salt (**C**) groups. In TNBS and TNBS+salt colon sections, fibrosis (arrow) led to neutrophil infiltrate and collagen deposits (magnification: 10×). Histologic inflammatory score from 0 (no inflammation) to 3 (severe inflammation) (**D**). Histologic fibrosis score from 0 (no fibrosis) to 3 (severe fibrosis) (**E**). One-way ANOVA followed by Tukey post-test: *** means p<0.001.


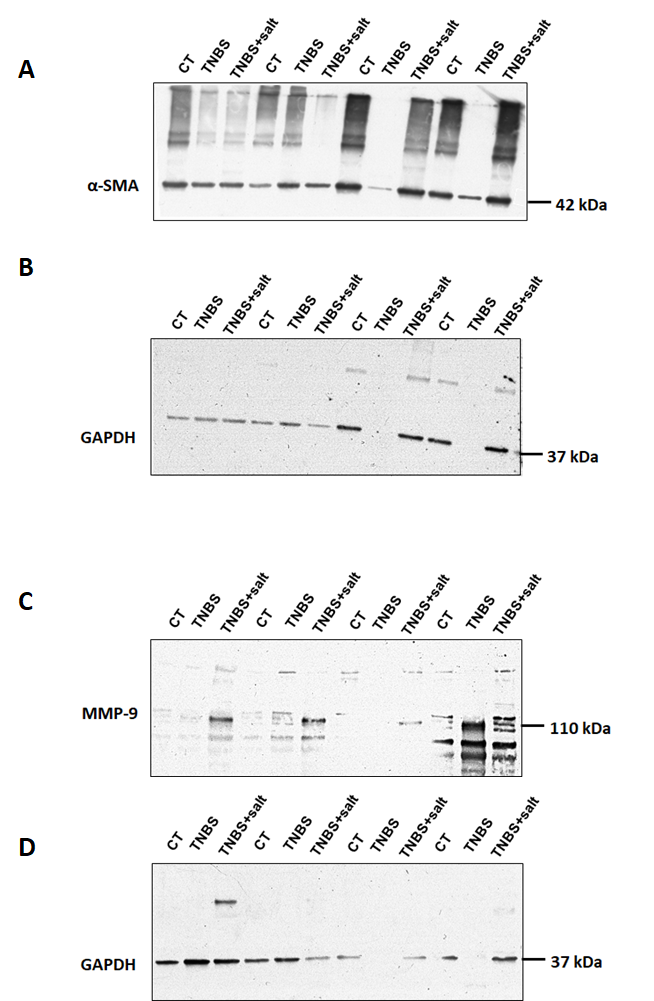


**Supplementary figure 2. Representative Western Blot image in colon of chronic TNBS-induced colitis rats.**

*α-sma* (alpha smooth muscle actin) (**A**) and its corresponding housekeeping GAPDH (**B**).

MMP-9 (matrix metalloproteinases) (**C**) and its corresponding housekeeping GAPDH (**D**)*.*


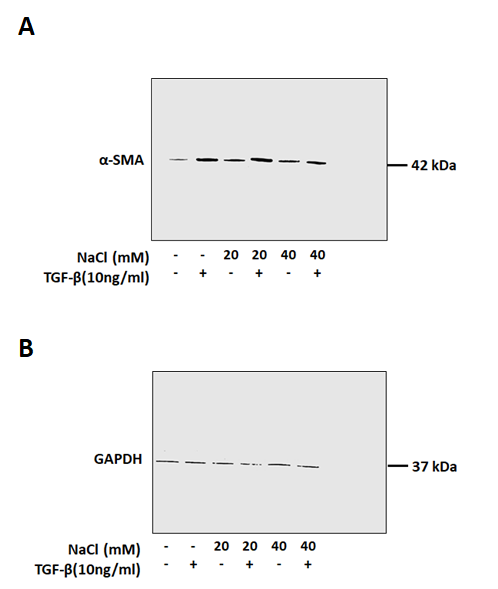


**Supplementary figure 3.** **Representative Western Blot image in CCD-18co cells lysates.**

*α-sma* (alpha smooth muscle actin) (**A**) and its corresponding housekeeping GAPDH (**B**).


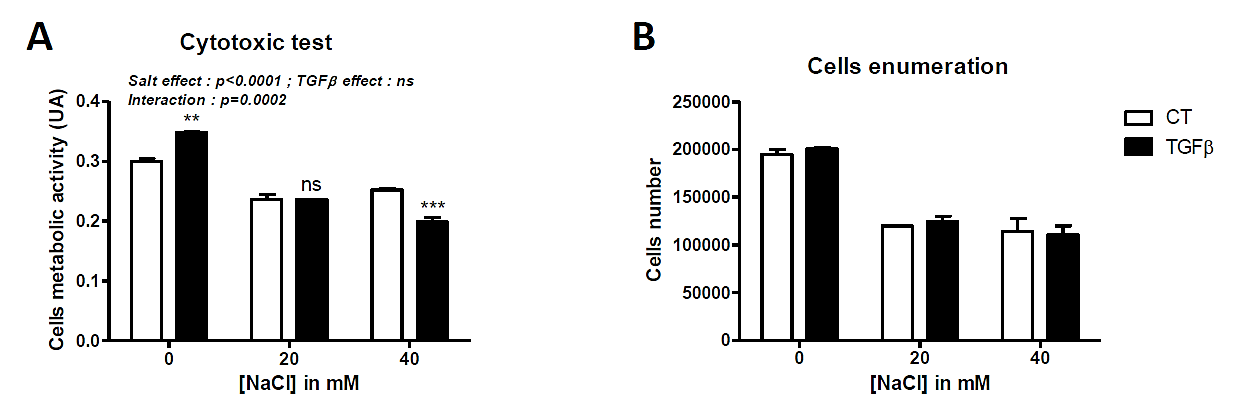


**Supplementary figure 4**

**NaCl treatment induces cytotoxic response in CCD-18co in response to TGF-β for 24h.**

Cells metabolic activity (**A**) and cell number by Malassez cell (**B**).

CCD-18Co cells were incubated with increasing concentration of dietary salt (0, 20 and 40 mM) in response to TGF-β (10ng/mL) for 24h. (n=3 from independent experiments). Two-way ANOVA test followed Bonferroni post-test: **means p<0.01, ***p<0.001 respectively.
